# Case Report: Herpes simplex virus type 2 (HSV-2) meningo-encephalitis associated with traumatic brain injury – a case report from Lao PDR

**DOI:** 10.12688/wellcomeopenres.22720.1

**Published:** 2024-08-20

**Authors:** Souphaphone Vannachone, Anouphet Chanthamavong, Malavanh Vongsouvath, Phetkim Sayasene, Manivanh Vongsouvath, Audrey Dubot-Pérès, Elizabeth A. Ashley, Terry John Evans

**Affiliations:** 1 Mahosot Hospital, Lao-Oxford-Mahosot Hospital-Wellcome Trust Research Unit, Vientiane, Vientiane Prefecture, Lao People's Democratic Republic; 2 Infectious Diseases Department, Mittaphab Hospital, Vientiane, Lao People's Democratic Republic; 3 Microbiology Department, Mahosot Hospital, Vientiane, Vientiane Prefecture, Lao People's Democratic Republic; 4 Unité Des Virus Emergents, Aix Marseille University, Marseille, France; 5 Nuffield Department of Medicine, University of Oxford Centre for Tropical Medicine and Global Health, Oxford, England, UK

**Keywords:** Herpes simplex virus, viral reactivation, meningitis, encephalitis, head injury, aciclovir, LMICs

## Abstract

**Background:**

Neurological symptoms following head trauma are common; however, the cause may not always be obvious. In the absence of open wounds, fractures, or surgical interventions, infectious causes may not be considered, and investigations may not be targeted to investigate this possibility.

**Case:**

A 39-year-old male presented with a severe headache, reduced consciousness, and confusion. Two days earlier, he had been discharged from the hospital, where he had been treated for traumatic brain injury with subarachnoid hemorrhage following a road traffic accident. Herpes simplex virus type 2 (HSV-2) was detected in the cerebrospinal fluid, confirming the diagnosis of viral meningoencephalitis. He was treated with oral aciclovir for two weeks and achieved full neurological recovery.

**Conclusions:**

This case highlights the risk of viral reactivation following trauma, particularly head injuries. Central nervous system infections should be considered in cases of delayed deterioration following trauma. The optimal treatment of herpes simplex virus (HSV) encephalitis may be challenging in resource-limited settings.

## Plain language summary

Herpes simplex virus type 2 (HSV-2) is a common virus that many people are infected by. It causes genital and orolabial herpes (also known as cold sores). It can also rarely cause infections of the central nervous system. Once a person is infected, the virus remains in the body permanently. The infection is normally dormant, however, the virus can reactivate periodically, and cause recurrent symptoms. Re-activation may occur if the person is unwell or if the body is stressed. Sometimes, the cause for reactivation is not obvious. In this case, serious head trauma caused reactivation of HSV-2, and this led to an infection of the lining of the brain, and perhaps of the brain itself. HSV-2 infection of the brain is life-threatening, and requires intravenous medication - aciclovir. In low-resource settings, the diagnostic tests and treatments required for HSV-2 are not readily available, and they may be too expensive for many people to afford. Therefore, we must increase diagnostic capacity where needed, as well as access to life-saving medications.

## Introduction

Herpes simplex virus (HSV) infections of the central nervous system (CNS) include overlapping but distinct syndromes of meningitis, meningoencephalitis, and encephalitis. They are among the most severe viral infections of the brain; untreated mortality is around 70% for HSV encephalitis, but this is reduced to 15% with timely treatment with intravenous aciclovir. Various other neurological manifestations of HSV infection have been described ^1^.

While meningitis and encephalitis are distinct clinical syndromes, their symptoms may overlap. For example, the cardinal features of meningitis (fever, headache, neck stiffness, and photophobia) may result in drowsiness, even without brain parenchymal involvement. In contrast, encephalitis requires altered consciousness including drowsiness, behavioral changes or coma, and focal neurological deficits may also be seen. While challenging, it is important to distinguish between these two syndromes because their treatment and prognosis differ. In particular, there is no convincing evidence that viral meningitis benefits from antiviral therapy, whereas encephalitis does.

HSV is the second most common cause of viral meningitis in adults after enterovirus ^2^ and is the most common cause of viral encephalitis in developed countries ^3^. The aetiology of viral encephalitis in tropical countries differs considerably, with Japanese encephalitis virus and dengue virus infections being particularly common in children ^4^, despite being vaccine-preventable.

Most cases of encephalitis caused by HSV in adults and children are due to HSV type 1 (~90%). HSV-2 is more often seen in HSV encephalitis in neonates (due to its association with genital herpes) and in immunocompromised patients ^5^ and may be seen in meningitis in all patient groups.

Delays in diagnosis and difficulties in providing optimal care are responsible for much of the considerable mortality and morbidity associated with these infections ^2, 6, 7^, and this is particularly problematic when cerebrospinal fluid (CSF) analysis is not possible. For example, in Laos, lumbar puncture and CSF analysis are available only in Vientiane Capital, and microbiological analysis (including viral polymerase chain reaction (PCR)) is offered in a single hospital laboratory. A delay in aciclovir administration is a key modifiable risk factor for poor outcomes ^8^.

Not only is significant mortality seen early in HSV encephalitis, but significant long-term cognitive, psychiatric, and neurodevelopmental sequelae are also very common ^9^. For example, 50% of survivors in a French cohort had moderate or severe disability as measured by the Glasgow Outcome Scale after 1 year ^10^, and the risk of epilepsy is significantly increased ^11^. Such studies of HSV encephalitis outcomes have not been conducted in Laos, but a recent detailed study discovered that the health and financial sequelae of Japanese encephalitis in Laos and Vietnam are often catastrophic ^12^.

Both HSV-1 and HSV-2 infections are very common, although many people may not experience the classic presentation of orolabial or genital herpes. The seroprevalence of HSV-1 is usually reported as 60–90%, while the seroprevalence of HSV-2 in the general adult population of Europe is approximately 12%, similar to the seroprevalence in Asia ^13, 14^. HSV encephalitis is caused by viral reactivation in approximately 70% of cases. Thus, while HSV encephalitis is uncommon, a significant proportion of the population is at risk of an underlying infection.

Although HSV meningitis and encephalitis are well-known syndromes, their association with head trauma has not been described previously. This case report highlights the reactivation of HSV-2 after traumatic brain injury and discusses the difficulties in managing this life-threatening infection in low-resource settings.

## Case presentation

A 39-year-old male was admitted with severe headache, drowsiness, confusion, and reduced consciousness. There was no fever, vomiting, or seizures. He had been discharged from the hospital two days earlier following a road traffic accident that caused traumatic brain injury, and a CT scan showed subarachnoid hemorrhage. The admission had lasted nine days, after which he had largely recovered.

The patient’s medical history was otherwise unremarkable, although he was a cigarette smoker and regularly used amphetamines. His HIV test on admission was negative.

On admission, vital signs showed mild hypertension (149/96 mmHg) and tachycardia (heart rate, 188 beats per minute). Peripheral blood leucocyte count was elevated at 15.97 x 10 ^3^ cells/µL (normal range 6.00–8.00 x 10 ^3^/µl), with a neutrophilia (12.8 x10 ^3^/µl) and a marked hyponatraemia of 125 mmol/L (normal range 136–145 mmol/L). His Glasgow Coma Scale score (GCS) was 14/15 (E3, V5, M6). The patient received fluid resuscitation and supplemental sodium chloride intravenously.

An initial review by the neurology team shortly after admission was normal, but on subsequent review the same day, the patient was found to have left hemiplegia (power 3/5 in the left arm), and his GCS had again dropped to 14 (E3, V5, M6), now with possible neck stiffness. Further treatment was initiated with ceftriaxone (2 g intravenously, twice daily), dexamethasone (8 mg orally, three times daily), and diazepam as empirical treatment for bacterial meningitis.

A repeat CT scan of the brain was performed, and the recurrence of intracranial hemorrhage and other acute abnormalities were excluded.

A lumbar puncture (LP) was performed the following afternoon (day 2 of admission), demonstrating a white cell count of 390/mm ^3^ (95% lymphocytes). The glucose level in the cerebrospinal fluid was 38 mg/dL (paired peripheral blood glucose was 145 mg/dL), protein was 72 mg/dL, the opening pressure was not measured, India ink stain for *Cryptococcus* spp. was negative, and rickettsial rapid diagnostic tests for murine (GenBio ImmunoDOT) and scrub typhus (IgM, InBios) were also negative. No organisms were seen on Gram staining, and no bacterial growth was observed on chocolate agar. A viral PCR panel was planned; however, the results were not immediately available.

On day 3 of admission, the patient was alert, with a GCS score of 15/15 and full power in all limbs, but with ongoing neck stiffness. Over the coming days, the persistent headache began to improve slowly.

On the sixth day of admission, HSV-2 was detected in the CSF by real-time PCR. Oral aciclovir 800 mg four times a day was added to treat HSV meningoencephalitis, and in the absence of MRI scanning, it was difficult to determine whether the patient had meningitis or encephalitis, although changes to consciousness or neurology were brief. No skin lesions suggestive of herpes were noted, and we were not able to ascertain whether the patient had a history of orolabial or genital herpes. Electrolytes were re-measured, and sodium levels were normal (136 mmol/L).

On day 10 of admission, the patient developed pedal oedema and diplegia, likely due to immobility and reduced albumin (3.0 g/dL; ref range 3.5–5.2 g/dL). By day 12, the patient had fully recovered, and ceftriaxone, oral aciclovir, and dexamethasone were discontinued. Repeat LP was not performed owing to financial constraints.

## Discussion

The patient was diagnosed with HSV-2 meningoencephalitis. However, in retrospect, the clinical features were most consistent with meningitis. The observed reduction in consciousness was minimal (the lowest GCS score recorded was 14/15), and meningitis associated with marked hyponatraemia was sufficient to explain this. Furthermore, the patient’s neurological status rapidly normalized several days before administration of aciclovir, a clinical course that is not typical of untreated HSV encephalitis.

CNS infections following head trauma may be suspected when there are physical injuries, where bacterial and fungal pathogens can be directly inoculated into the central nervous system. However, in the absence of fractures or penetrating wounds, infections are considered less likely. Consequently, the infectious aetiology of our patient’s re-presentation was unexpected, highlighting the importance of maintaining a broad differential diagnosis. Early lumbar puncture is important when safe to perform, but this is seldom achieved even in high-income settings ^2^. Other possible infectious causes in this case include viruses and bacteria, including *Mycobacterium tuberculosis*, and fungi such as *Cryptococcus* spp. Differential diagnosis includes metabolic derangement (e.g., hyponatraemia, hypoglycaemia), toxins (including recreational drugs), and intracranial thrombosis.

In 1978, reactivation of HSV-1 following skin trauma was demonstrated in mice ^15^. However, reactivation of herpesviruses, both HSV and Varicella zoster virus (VZV), following trauma is not frequently considered in clinical practice. Despite this, using a case-control study design of 16,771 cases, herpes zoster (shingles) was shown to be 3.4 times more common in patients who had experienced trauma in the previous 7 days (95% CI 2.8-4.2), and remarkably was 27.5 times more common (95% CI 5.4-140.3) if the injury was to the head ^16^. In one case, a minor head injury was followed by a disseminated vesicular rash, most marked on the forehead, and the vesicle fluid tested positive for both VZV and HSV-1 ^17^. These cases of reactivation involve the skin, but viral reactivation leading to CNS infection is less commonly described but has occurred following neurosurgery ^18^; thus, our case is an important reminder of this possibility. The mechanism by which some triggers may result in HSV reactivation is understood ^19^, and various data support a mechanistic explanation for immune dysfunction following head injury ^20^. Notably, the pro-inflammatory response following traumatic brain injury alters immune cell behavior as well as cytokine production as part of a sequence of events that increase host susceptibility to infection.

Clinical guidelines unambiguously mandate treatment with intravenous aciclovir 10 mg/kg three times a day for HSV encephalitis if initial CSF analysis suggests viral infection ^21^, although in our case, treatment was only started once a positive PCR result was obtained on day 6 of admission. The recommendation is usually for 14–21 days of intravenous therapy, stopping only when the repeat CSF analysis is negative for HSV by PCR. Given our patient’s full clinical recovery and the costs associated with repeat lumbar punctures, he did not undergo repeat CSF analysis.

Achieving standards set by international guidelines may be aspirational in some settings, especially in low-income and middle-income countries. Intravenous aciclovir is not on the lists of essential medicines produced either in Laos or by the World Health Organization ( https://iris.who.int/bitstream/handle/10665/371090/WHO-MHP-HPS-EML-2023.02-eng.pdf?sequence=1), and it is very rarely used in Laos. This situation is common in many LMICs ^22^. Patients are instead treated with the oral formulation, which has a bioavailability of only 15–20%, and variable CSF penetration ^23^. Consequently, clinical outcomes are likely to be much poorer than those in settings in which intravenous treatment is the standard of care.

The oral prodrug of aciclovir, valaciclovir, has superior bioavailability and has been proposed as an option when intravenous aciclovir is unavailable. A 2011 study demonstrated that adequate CNS concentrations of aciclovir were achieved when valaciclovir (1 g) was administered three times daily ^24^. However, the use of valaciclovir is far from settled, and a higher dosing regimen of 2 g three times a day or even four times a day has been proposed more recently, based on pharmacokinetic and pharmacodynamic considerations ^23^. Valaciclovir is not available in Laos.

Despite these theoretical and practical concerns, oral aciclovir has some efficacy in the treatment of HSV encephalitis. Furthermore, a report of HSV encephalitis successfully treated with a 10-day course of oral aciclovir emphasized the 28-fold cost differential between intravenous and oral treatment ^25^, which is also a key consideration in the Lao context, where healthcare expenses are frequently catastrophic ^12, 26^.

This study has some limitations. HSV encephalitis (especially HSV-1) typically involves the temporal lobes, and MRI is often performed to confirm the diagnosis; however, this was not performed in this case. Second, while we believe that the clinical presentation was due to viral reactivation, which is the most common scenario, we cannot exclude primary infection; measuring type-specific antibodies in the serum can be performed to clarify this point, but this test was not available and would not have altered clinical management.

## Conclusions

This case highlights the growing recognition that trauma can cause reactivation of herpes viruses, including reactivation in the central nervous system, and that infectious complications are important in the differential diagnosis of neurological complications following head trauma. Furthermore, we highlight the lack of access to life-saving antiviral drugs in many countries along with the financial and practical challenges associated with managing these infections in many settings.

## Public and patient involvement

There was no formal patient or public involvement in the design or conduct of the study.

## Ethic and consent

Written informed consent was obtained from the patient for the publication of this case report.

## Data Availability

No data are associated with this article.
