# Supplementary material for: Case Report: Herpes simplex virus type 2 (HSV-2) meningo-encephalitis associated with traumatic brain injury – a case report from Lao PDR
Source: Wellcome Open Res. 2025 Dec 4;9:489. Originally published 2024 Aug 20. [Version 2] doi: 10.12688/wellcomeopenres.22720.2 (PMC12715399; doi:10.12688/wellcomeopenres.22720.2)
Supplement: Peer review history

# Reviewer report: Reviewer response for version 2

**DOI:** 10.21956/wellcomeopenres.28129.r141561

**Published:** 2025-12-18

**Authors:** Brent Stanfield

**Affiliations:** 1 Louisiana State University, Baton Rouge, USA

The updated manuscript is appropriate for publication.

Are enough details provided of any physical examination and diagnostic tests, treatment given and outcomes?

Yes

Is the case presented with sufficient detail to be useful for other practitioners?

Yes

Is sufficient discussion included of the importance of the findings and their relevance to future understanding of disease processes, diagnosis or treatment?

Partly

Is the background of the case’s history and progression described in sufficient detail?

Yes

Reviewer Expertise:

I focus on preclinical herpesvirus vaccine development, herpesvirus molecular virology, and immunopathogenesis, aiming to translate findings into innovative therapeutic and prophylactic vaccines.

I confirm that I have read this submission and believe that I have an appropriate level of expertise to confirm that it is of an acceptable scientific standard.

# Reviewer report: Reviewer response for version 2

**DOI:** 10.21956/wellcomeopenres.28129.r141560

**Published:** 2025-12-18

**Authors:** Franklyn Nkongho Egbe

**Affiliations:** 1 University of Liverpool, Liverpool, UK

The authors have satisfactorily responded to my comments.  I have no further comments to make.

Are enough details provided of any physical examination and diagnostic tests, treatment given and outcomes?

Yes

Is the case presented with sufficient detail to be useful for other practitioners?

Partly

Is sufficient discussion included of the importance of the findings and their relevance to future understanding of disease processes, diagnosis or treatment?

Partly

Is the background of the case’s history and progression described in sufficient detail?

Yes

Reviewer Expertise:

HSV diagnosis

I confirm that I have read this submission and believe that I have an appropriate level of expertise to confirm that it is of an acceptable scientific standard.

# Reviewer report: Reviewer response for version 1

**DOI:** 10.21956/wellcomeopenres.25022.r137463

**Published:** 2025-11-21

**Authors:** Franklyn Nkongho Egbe, Abdusshakur Muhammad Auwal

**Affiliations:** 1 University of Liverpool, Liverpool, UK; 2 University of Liverpool Institute of Infection Veterinary and Ecological Sciences, Neston, England, UK

**Overall Summary**

 Thank you for inviting me to review this case report entitled “Herpes simplex virus type 2 (HSV-2) meningoencephalitis associated with traumatic brain injury – a case report from Lao PDR” by Vannachone and colleagues . The case report highlights a case of HSV infection - probably meningoencephalitis – likely caused by HSV-2 reactivation following traumatic brain injury (TBI) in a resource-limited setting with limited access to laboratory diagnosis of CNS infections and antivirals.  

 The case is a 39-year-old male patient discharged from the hospital after being treated for TBI following a road traffic accident. He was readmitted 9 days later with severe headache, drowsiness, confusion and reduced consciousness. After 6 days of unsuccessful empirical treatment for bacterial meningitis with ceftriaxone (2 g intravenously, twice daily), dexamethasone (8 mg orally, three times daily), and diazepam, HSV-2 was detected in his CSF by PCR.  Oral aciclovir, 800 mg, was then added to his treatment regimen. The patient fully recovered by day 12, and treatment was discontinued. The authors suggested that HSV CNS infections should be considered in cases of delayed deterioration following TBI and further highlighted the challenges of optimal HSV infection treatment in resource-limited settings.

**Overall Comments**

 The authors have provided a detailed description of the cases’ history and progression, the clinical examination and diagnostic tests used, and the treatment and clinical outcome.

 However, they could add detailed information on the clinical features to guide other practitioners (see detailed comments below), and some recommendations for future research. Also, are there any clinical findings from his first admission (similar to those presented here) that could be included? e.g. it could be interesting to compare his first and second admissions' sodium levels and GCS scores.

**Points to address to make the article scientifically sound**

**Abstract**:

**Background**: Could the authors rephrase the background paragraph to focus more on the topic: HSV-2 CNS infections

*Text…Central nervous system infections should be considered in cases of **delayed deterioration** following trauma…*

 Could the authors include some specific clinical picture of ‘ *delayed deterioration’* from this case to guide other practitioners, e.g. severe headache, drowsiness, confusion, reduced consciousness, hyponatraemia and/or neurological deficits, as detailed in the case presentation?

**Introduction:**

 - Could the authors describe previous case reports or studies on Traumatic brain injury and HSV infection? E.g. Bhimani et al 2024

 - Could the authors add a brief review on CNS infection from HSV reactivation? e.g. what are the likely causes of HSV reactivation, and is TBI normally considered one of the causes? Could they provide information to enhance the reader's understanding of primary and reactivated HSV CNS infection?

 Text : *Although HSV meningitis and encephalitis are well-known syndromes, their association with head trauma has not been described previously*

 What about the case report by Bhimani et al 2024?

*Text: ….This case report highlights the reactivation of HSV-2 after traumatic brain injury and discusses the difficulties in managing this life-threatening infection in low-resource settings….*

 Could the authors rephrase the objective to match their case report? E.g.  … a case report of CNS infection caused by HSV-2 reactivation following TBI…?

**Discussion**:

*Text…..although in our case, treatment was only started once a positive PCR result was obtained on day 6 of admission…*

 If there was clinical suspicion of meningoencephalitis from onset, why was aciclovir treatment delayed?

**Conclusion**

*Text: …highlights the growing recognition that trauma can cause reactivation of herpes viruses, including reactivation in the central nervous system, and that infectious complications are important in the differential diagnosis of neurological complications following head trauma…*

 The authors could rephrase this to highlight that TBI could cause HSV-2 reactivation, leading to CNS infection, and thus should be considered in the differential diagnosis of neurological complications following TBI.

 Could the authors suggest future studies to enhance our understanding of HSV infections caused by HSV reactivation following TBI?

Are enough details provided of any physical examination and diagnostic tests, treatment given and outcomes?

Yes

Is the case presented with sufficient detail to be useful for other practitioners?

Partly

Is sufficient discussion included of the importance of the findings and their relevance to future understanding of disease processes, diagnosis or treatment?

Partly

Is the background of the case’s history and progression described in sufficient detail?

Yes

Reviewer Expertise:

HSV diagnosis

We confirm that we have read this submission and believe that we have an appropriate level of expertise to confirm that it is of an acceptable scientific standard, however we have significant reservations, as outlined above.

## References

[1] : A rare case of herpes simplex virus encephalitis from viral reactivation following surgically treated traumatic brain injury. *Brain Injury* .2024;38(12) : 10.1080/02699052.2024.2370834 1046-1051 10.1080/02699052.2024.2370834 38963013

# Response

**Published:** 2025-12-01

**Authors:** Souphaphone VANNACHONE

**Affiliations:** Microbiology laboratory, Lao Oxford Mahosod Hospital Welcome trust Research Unit, Vientiane capital, Lao People's Democratic Republic

We thank the Reviewer for for their thoughtful comments on our article. We have attempted to address each of these, as described below.

**Comment: **1. Are there any clinical findings from his first admission?

**Response: **Unfortunately, the medical records are no longer available, and therefore we are unable to provide blood test results from the initial admission.

**Comment: **2. Rephrase the background paragraph to focus more on the topic: HSV-2 CNS infections          

**Response: **We have now specifically emphasised the possibility of viral infections in the Abstract.

**Comment: **3. Include some specific clinical picture of ‘ *delayed deterioration’.* 

**​​​​​​​Response: **We have added the typical symptoms of worsening headache, drowsiness and reduced cognitive state in this paragraph.

**Comment: **4. Describe previous case reports or studies on Traumatic brain injury and HSV infection? E.g. Bhimani et al 2024.

**​​​​​​​Response: **Thank you highlighting this recent article, which we have now included.

**Comment: **5. Add a brief review on CNS infection from HSV reactivation. e.g. what are the likely causes of HSV reactivation, and is TBI normally considered one of the causes?

**​​​​​​​Response: **Details on the mechanisms of HSV reactivation are now provided, including loss of host-derived proteins that maintain viral latency, local immune dysfunction involving pro-inflammatory microglial activation, and systemic effects via sympathetic activation and glucocorticoid release. Citations are added to support this new information.

**Comment: **6. Could they provide information to enhance the reader's understanding of primary and reactivated HSV CNS infection?

**​​​​​​​Response: **The difficulty in distinguishing between primary infection and reactivation on clinical grounds alone is stated explicity, adding that IgG antibody testing can distinguish between the two possibilities.

**Comment: **7. Rephrase the objective to match their case report? E.g.  … a case report of CNS infection caused by HSV-2 reactivation following TBI…?

**​​​​​​​Response: **We have re-focused this sentence by re-phrasing it as suggested: “Our case report highlights CNS infection caused by HSV-2 reactivation after traumatic brain injury in the absence of surgery and discusses the difficulties in managing this life-threatening infection in low-resource settings.”

**Comment: **8. Why was aciclovir treatment delayed?

**​​​​​​​Response: **Viral infection was not anticipated, although we recognise a lymphocytic CSF should have raised clinical suspicion sooner. In addition, aciclovir is very expensive in Laos and empiric prescription is uncommon.

**Comment: **9. Rephrase this to highlight that TBI could cause HSV-2 reactivation,

**​​​​​​​Response: **We have re-written the text to emphasise that traumatic brain injury specifically may cause reactivation as follows: “This case highlights the growing recognition that traumatic brain injury causes reactivation of herpes viruses, including reactivation in the central nervous system, and that infectious complications are important in the differential diagnosis of neurological complications following traumatic brain injury.”

**Comment: **10. Suggest future studies to enhance our understanding of HSV infections caused by HSV reactivation following TBI. 

**​​​​​​​Response: **​​​​​​​We have suggested an observational study in the first instance whereby CSF is routinely tested for viruses, and serum HSV IgG titres measured. Neurosurgical centres are likely to have suitable patient numbers and expertise.

# Reviewer report: Reviewer response for version 1

**DOI:** 10.21956/wellcomeopenres.25022.r112708

**Published:** 2024-12-05

**Authors:** Brent Stanfield

**Affiliations:** 1 Louisiana State University, Baton Rouge, USA

The article, titled *"Herpes simplex virus type 2 (HSV-2) meningoencephalitis associated with traumatic brain injury – a case report from Lao PDR,"* describes a compelling case of a 39-year-old male who experienced neurological symptoms, including severe headache and altered consciousness, following traumatic brain injury (TBI). After a road traffic accident resulting in subarachnoid hemorrhage, the patient initially recovered but later presented with symptoms indicative of meningoencephalitis. Diagnostic testing ultimately revealed HSV-2 in the cerebrospinal fluid (CSF), confirming viral meningoencephalitis. Treated with oral acyclovir, the patient achieved full neurological recovery. This case report offers valuable insights into the rare association between TBI and HSV-2 reactivation and highlights the challenges of diagnosing and treating such cases in resource-limited settings.

 The background of the patient’s history and progression is described in reasonable detail. However, some aspects could benefit from further clarification. The authors provide a thorough account of the initial trauma and subsequent hospital admission, but the timeline of events following discharge could be expanded to better illustrate the progression of symptoms. For instance, specifying whether the neurological symptoms emerged gradually or suddenly after discharge would enhance the understanding of how HSV-2 reactivation was triggered by trauma. This information would be particularly useful for practitioners who might encounter similar cases.

 Details of the physical examinations, diagnostic tests, treatments, and outcomes are generally comprehensive. The authors describe the patient’s clinical presentation, initial diagnostic workup, and the decision to treat empirically for bacterial meningitis while awaiting viral PCR results. The diagnostic process, including CSF analysis and PCR confirmation of HSV-2, is well-documented. However, there are opportunities to strengthen the discussion around the treatment decisions. The authors note the use of oral acyclovir due to resource limitations but do not sufficiently elaborate on the clinical implications of using oral rather than intravenous acyclovir, which is the standard of care for HSV meningoencephalitis. This omission leaves the reader questioning how these constraints may have influenced the patient’s recovery trajectory and whether alternative antiviral therapies, such as valacyclovir, were considered or feasible in this setting. Additionally, further context regarding the significance of the elevated leukocyte count and hyponatremia observed in the patient would provide a more nuanced understanding of the clinical findings.

 The discussion of the findings is thought-provoking and highlights the novelty of the case, particularly the link between TBI and HSV reactivation. The authors effectively emphasize the importance of maintaining a broad differential diagnosis when neurological symptoms develop following trauma. They also draw attention to the diagnostic and therapeutic challenges posed by resource limitations in low-income settings. Nevertheless, the discussion would benefit from a deeper exploration of the mechanisms by which trauma may trigger HSV-2 reactivation. While the authors briefly touch on immune dysregulation as a potential factor, they could enhance their argument by integrating evidence from existing literature or animal models that demonstrate the pathways leading to viral reactivation after trauma. Such an expansion would strengthen the scientific foundation of the case report and its broader implications for understanding HSV pathogenesis.

 The report is presented with sufficient detail to be useful to other practitioners, particularly those working in resource-limited environments. It provides actionable insights into the diagnostic approach and treatment options for similar cases. However, the article would be even more practical if it included a concise summary of lessons learned or key recommendations. For example, a brief reflection on how clinicians can navigate the challenges of limited diagnostic tools and treatments would make the report more accessible and relevant to a global audience.

 To ensure the article is scientifically sound, the authors should address several key points. They should clarify the timeline of symptom progression to provide a clearer picture of the case’s trajectory. The rationale for choosing oral acyclovir should be discussed in greater detail, along with a reflection on how this decision aligns with international treatment guidelines and its implications for patient outcomes. Expanding the discussion on the mechanisms of HSV-2 reactivation would also enhance the scientific rigor of the report. Lastly, summarizing actionable insights for practitioners would improve the utility of the case report.

 In summary, the article is a valuable contribution to the literature, shedding light on an unusual but clinically significant association between TBI and HSV-2 reactivation. While it is largely well-written and informative, addressing the points above will strengthen its clarity, scientific depth, and practical relevance. With these revisions, the report will serve as an excellent resource for clinicians and researchers alike.

Are enough details provided of any physical examination and diagnostic tests, treatment given and outcomes?

Yes

Is the case presented with sufficient detail to be useful for other practitioners?

Yes

Is sufficient discussion included of the importance of the findings and their relevance to future understanding of disease processes, diagnosis or treatment?

Partly

Is the background of the case’s history and progression described in sufficient detail?

Yes

Reviewer Expertise:

I focus on preclinical herpesvirus vaccine development, herpesvirus molecular virology, and immunopathogenesis, aiming to translate findings into innovative therapeutic and prophylactic vaccines.

I confirm that I have read this submission and believe that I have an appropriate level of expertise to confirm that it is of an acceptable scientific standard, however I have significant reservations, as outlined above.

# Response

**Published:** 2025-12-01

**Authors:** Souphaphone VANNACHONE

**Affiliations:** Microbiology laboratory, Lao Oxford Mahosod Hospital Welcome trust Research Unit, Vientiane capital, Lao People's Democratic Republic

We are grateful to the Reviewer for their careful reading of our manuscript. We have endeavoured to answer all suggestions, which we agree strengthen our article, and we provide a point-by-point response.  

**Comment: **1. The timeline of events following discharge could be expanded.

**Response:** The readmission was only 2 days after discharge, so not a significant timeframe for complex new symptomatology to evolve. We have emphasised the gradual worsening of symptoms, which were non-specific.

**Comment: **2. Elaborate on the clinical implications of using oral rather than intravenous acyclovir … was valacyclovir considered or feasible? The text states that intravenous aciclovir is the only recommended option for herpes simplex encephalitis.

**Response: **We have stated that use of oral aciclovir may lead to worse outcomes due to pharmacokinetic parameters; and we have added that this has not been studied in trials. We have described the likely inferior role of valacyclovir, and we have explained that valacyclovir is not available in Laos.

**Comment: **3. Significance of the elevated leukocyte count and hyponatremia.

**​​​​​​​Response: **The updated text explains that these may have various causes, but following brain injury, may be due to inflammation and SIADH – although bacterial co-infection and hypovolaemia remain possible.

**Comment: **4. The mechanisms by which trauma may trigger HSV-2 reactivation.

**​​​​​​​Response: **Details on the mechanisms of HSV reactivation are provided, including loss of host-derived proteins that maintain viral latency, local immune dysfunction involving pro-inflammatory microglial activation, and systemic effects via sympathetic activation and glucocorticoid release. Citations are added to support this new information.

**Comment: **5. A concise summary of lessons learned or key recommendations.

**​​​​​​​Response: **We have provided the key lesson that immediate intravenous aciclovir is required when viral encephalitis is suspected clinically or suggested by CSF analysis.
